# Effect of Crop Type Shift on Soil Phosphorus Morphology and Microbial Functional Diversity in a Typical Yellow River Irrigation Area

**DOI:** 10.3390/microorganisms13071458

**Published:** 2025-06-23

**Authors:** Zijian Xie, Rongbo Zhao, Bo Bo, Chunhua Li, Yang Wang, Yu Chu, Chun Ye

**Affiliations:** 1 National Engineering Laboratory for Lake Pollution Control and Ecological Restoration, Chinese Research Academy of Environmental Sciences, Beijing 100012, China; xiezijian@craes.org.cn (Z.X.); 13841569400@163.com (R.Z.);; 2 Key Laboratory of Drinking Water Source Protection of the Ministry of Ecology and Environment, Chinese Research Academy of Environmental Sciences, Beijing 100012, China; 3 Faculty of Science, The University of Queensland, Brisbane 4101, Australia

**Keywords:** Yellow River irrigation area, soil phosphorus, crop type change, phosphorus morphology, phosphate-solubilizing microorganisms

## Abstract

The Hetao irrigation area is one of the largest irrigation areas in the Yellow River Basin and a typical salinized agricultural area. Crop type shifts in this area can alter soil phosphorus (P) morphology and microbial functional diversity, thereby influencing soil P losses. However, few studies have elucidated the underlying mechanisms. In this study, soil samples were collected from four different crop planting areas: sunflower field (SF), corn field (CF), wheat land (WL), and vegetable and fruit land (VFL). Subsequently, the physicochemical properties, P fractions, and phosphate-solubilizing microorganisms (PSMs) were analyzed. The results indicated that when other lands shifted to SF, the soil pH increased significantly. Simultaneously, SOM, TN, and TP decreased significantly during the crop type conversion. Analysis of P fraction revealed that moderately active P, including NaOH-P_i_, NaOH-P_o_, and HCl-P_i_, were the dominant fractions in the tested soils. Among them, HCl-P_i_ was the major component of moderately active P. The soil P leaching change point in the tested are was 6.25 mg Olsen-P kg^−1^. The probabilities of P leaching in WL, VFL, CF, and SF were 91.7%, 83.8%, 83.8%, and 66.7%, respectively. Additionally, the sum of the relative abundances of the three PSMs in SF, VFL, WL, and CF were 8.81%, 11.88%, 8.03%, and 10.29%, respectively. The shift in crop type to SF exacerbated the soil degradation process. Both TP and residual P in the soil decreased. However, the NaHCO_3_ slightly increased, which may have been due to the increased abundance of *Thiobacillus* and *Escherichia*.

## 1. Background

Adequate soil phosphorus (P) is essential for achieving optimal crop yields. Although most soils have considerable total phosphorus (TP) stocks, only a small fraction (<1%) of the total inorganic and organic P is readily soluble and available for plant uptake [1,2]. Plant roots mainly absorb dissolved P in soil solution as phosphate ions, namely hydrogen phosphate (HPO_4_^2−^) and dihydrogen phosphate (H_2_PO_4_^−^) ions, and their forms depend on the soil solution pH [1,3]. Consequently, continuous replenishment of soil P is necessary to meet the demands of growing crops [2,4]. However, over-applying P fertilizers, whether chemical, organic, or a combination, can cause P to accumulate in the soil through dissolution or organic P mineralization [5,6]. This increases the risk of P loss through runoff and leaching. Notably, P loss from agricultural soils is a major environmental concern as its significantly contributes to water-body eutrophication [7,8]. Therefore, controlling P loss from soil to water is crucial for improving water environmental quality.

Characterizing soil P fractions is essential for understanding the mechanisms of soil P migration and transformation [9,10]. The Hedley P sequential extraction method is the most widely used technique for separating various forms of soil P [11,12]. This method classifies soil P into three main categories: active P, moderately active P, and stable P [10,12]. By evaluating the binding strength of P within the soil matrix, the Hedley method provides insights into the availability and stability of P fractions [9,12]. Thus, understanding the variable characteristics of P components helps assess soil P loss risk and implement effective regional P management strategies.

Soil microorganisms play a crucial role in the P cycle and are regarded as the dominant factor driving soil P turnover [13,14]. Generally, microorganisms that increase soil P availability by promoting organic P decomposition or inorganic P desorption are called phosphate-solubilizing microorganisms (PSMs). All bacteria, actinomycetes, and fungi include phosphate-solubilizing genera [13,15]. Acidolysis is the main way for PSMs to dissolve P. The organic acids produced by these microorganisms can block P adsorption sites in the soil or form complexes with cations on soil mineral surfaces, thus enhancing P availability [15,16]. Moreover, PSMs can decompose organic phosphates or mineralize organic phosphate salts by secreting acid or alkaline phosphatases, converting them into soluble P that plants can absorb and utilize [2,17]. Meanwhile, PSMs release substances such as CO_2_ through respiration, which lowers the pH value of the surrounding soil environment, thereby dissolving insoluble phosphate salts [2,15]. Therefore, clarifying the community structure of soil PSMs is highly significant for understanding the mechanisms of soil P migration and transformation.

Soil salinization significantly impacts crop growth and nutrient uptake. On one hand, common crops experience yield reduction because they struggle to absorb water in a high-salinity environment [18,19]. In contrast, salt-tolerant crops can sustain growth via osmotic regulation [18,19]. On the other hand, salinization causes soil compaction, reduces microbial activity, and hinders SOM decomposition, which, in turn, affects crop root development [18,19]. Therefore, cultivating salt-tolerant crops is an inevitable choice for agricultural development in saline-alkali regions.

The Hetao irrigation area in Inner Mongolia is one of the main grain-producing areas in the Yellow River Basin of China, characterized by a high level of soil salinization [20]. The soil salinization process in this region is mainly influenced by natural factors and anthropogenic irrigation practices. Natural factors involve high salinity in the parent material and high evaporation rates, while dominant anthropogenic factors are excessive flood irrigation and poor drainage infrastructure [20,21]. The area of salinized cultivated land in this district reaches 3.94 × 10^5^ hm^2^, accounting for 68.7% of the total cultivated land area [21]. The main crop types in this region are sunflower fields (SF), corn fields (CF), wheat lands (WL), and vegetable and fruit lands (VFL), which, together, make up over 95% of the total cultivated area. In the past decade, the SF area has increased rapidly from 38% to 56%, with an average proportion of 49%. Sunflowers are widely planted on saline-alkali land because of their well-developed root systems, strong antioxidant capacity, and salt tolerance genes [18,22]. As soil salinization has spread, many field crop types have been replaced by sunflowers [18]. This transition in crop types leads to changes in management practices such as irrigation and fertilization, which, in turn, alter the soil P availability and storage, modify the structure of soil microbial communities, and can lead to changes in the migration and transformation characteristics of soil P. However, there have been few studies on the impact of crop type changes on the soil P morphology and microbial functional diversity in the Hetao irrigation area.

In this study, we hypothesize that the shift to SF led to a change in P leaching risk due to reduced nutrient inputs and altered microbial communities. The aims of this study were (i) to analyze the soil P storage, availability, and fractions influenced by crop type conversion; (ii) to investigate the abundance of soil PSMs affected by crop type change; and (iii) to clarify the major driving factors of P transformation.

## 2. Materials and Method

### 2.1. The Study Area

The study area was situated in Wuyuan County, Bayannur City, a typical irrigated agricultural area. The region has a mid-temperate continental climate. The annual average temperature ranges from 3.7 °C to 7.6 °C, and the annual average rainfall is between 130 and 285 mm [20]. Additionally, the annual average evaporation rate is between 2030 and 3180 mm. The river water mainly comes from agricultural irrigation and drainage canals. The main irrigation periods are spring irrigation, summer irrigation, and autumn irrigation, concentrated from April to May, from June to September, and from October to November, respectively. These periods are critical for agricultural activities and have significant impact on the area’s hydrological cycle and soil moisture dynamics.

According to the Statistical Yearbook of Bayannur City in 2023 (http://tjj.bynr.gov.cn/, accessed on 20 March 2025), the crop cultivation area in Wuyuan County was 1.4 × 10^5^ hm^2^, accounting for nearly 20% of the total area in Byannur City. In the past decade, the crop-planting area in Wuyuan County increased by 12,113 hm^2^, with a growth rate of 9.2%. WL, CF, SF, and VFL were the main crops, accounting for 2%, 29%, 56%, and 5% of the cultivated land area in Wuyuan County, respectively, totaling 96%. The SF area increased rapidly from 38% to 56%, with an average proportion of 49%. WL and VFL decreased from 23.0% to 2.2% and from 6.8% to 4.8%, respectively. The planted area of CF remained stable at around 27.6%. SF was mostly shifted by WL and VFL, with crop changes occurring over a period of 5~10 years.

### 2.2. Sample Collection

**Figure 1:**
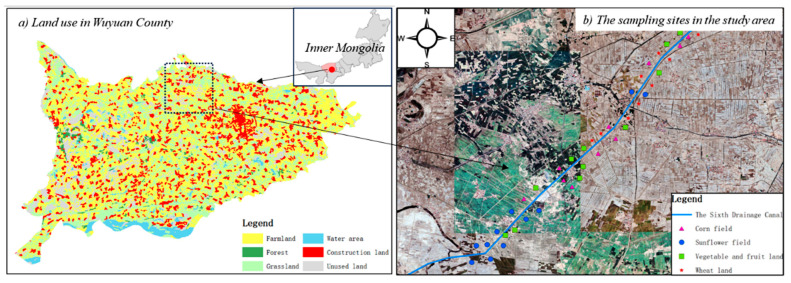
Land use and the sampling sites in the study area.

The soil type in the study was anthropogenic-alluvial soil. A sampling campaign was conducted in September 2023 in the sixth drainage basin (Figure 1). Soil samples were collected using a soil core sampler with 70 mm diameter. In total, 45 sampling sites were collected. At each site, three soil samples were taken and then mixed.

These sampling sites were spread across different crop types. There were 12 sites in SF and 11 sites each in WL, CF, and WFL. Based on the fertilization depth and plant root distribution, soil samples were collected from 0~20 cm layers and then transported to the laboratory in a refrigerator. The coordinates of the sampling points were recorded using GPS. Fresh samples were used for metagenomic analysis. Other samples were air-dried at room temperature and then sieved through 2 mm and 0.15 mm sieves for the determinations of other indicators.

### 2.3. Sample Analysis

A pH meter (S8-meter) was used to measure soil pH at a soil-to-water ratio of 1:2.5. The soil salinity was quantified by the gravimetric method. SOM was determined using the hydrated hot potassium dichromate oxidation-colorimetry method [10,23]. A laser particle size analyzer was used to analyze the soil particle size distribution (Mastersizer 2000, Malvern Panalytical Ltd., Malvern, UK). Total nitrogen (TN) content was assessed by the selenium powder-copper sulfate-potassium sulfate-concentrated sulfuric acid digestion-semi-micro Kjeldahl method [23].

Soil Olsen-P was extracted with 0.5 M NaHCO_3_ solution (pH = 8.5) at a soil-to-solution ratio of 1:20 [23,24]. CaCl_2_-P was extracted with a 0.01 M CaCl_2_ solution at a soil-to-solution ratio of 1:5 [24]. Total phosphorus (TP) was digested by HNO_3_-HF microwave and then determined by ICP-OES (Optima 5300DV, PerkinElmer, MA, USA). The P in the extracted or digested solution was determined by the molybdenum colorimetry method [23,24].

Soil P fractions were obtained using the Hedley sequential extraction procedure, which includes resin-P, NaHCO_3_-Pi, NaHCO_3_-Po, NaOH-Pi, NaOH-Po, HCl-Pi, and residual P [10,12]. The P in the extracted solution was also determined by the molybdenum colorimetry method [23,24]. Total dissolved phosphorus (Pt) in the filtrates was determined by the molybdenum colorimetry method after digestion with alkaline potassium persulfate [23,24]. Organic P (Po) was calculated as Pt minus Pi.

The quantitative metagenomics analysis was conducted by Wekemo Tech Group Co., Ltd. (Shenzhen, China). Genomic DNA was extracted from 0.25 g soil samples using the cetyltrimethylammonium bromide method [25,26,27]. The quality of the extracted DNA was evaluated using a NanoDrop 2000 spectrophotometer (Thermo Scientific, Wilmington, DE, USA) by measuring absorbance at 260 and 280 nm. DNA was quantified using a Qubit^®^ 2.0 Fluorometer (Thermo Scientific, Wilmington, DE, USA). Metagenomic sequencing libraries were built with the NEBNext Ultra II DNA Library Prep Kit for Illumina (E7370, NEB, MA, USA), and sequencing was carried out on an Illumina PE150 sequencer (Illumina, San Diego, CA, USA). Quality control of the sequencing reads was conducted using FastQC software [26,27]. For comparative analysis, Kraken 2 and a customized database were used for the taxonomic classification of species. Then, Bracken was used to predict the actual relative abundance of species in the samples [26,27].

### 2.4. Data Analysis

The data were analyzed and visualized using SPSS 27.0 and Origin 2022. After conducting the homogeneity of variance test and the normality test, a one-way ANOVA test and LSD test were carried out with SPSS 27.0 to assess the statistical significance among different crop types. The soil P leaching change point and its risk probability were calculated using the SPOLERC software [28]. The software can be accessed at https://github.com/FanZhang0830/SPOLERC, accessed on 20 March 2025. Pearson correlation analysis and linear regression modeling were performed using Origin 2022.

## 3. Results and Discussion

### 3.1. Effect of Crop Type Shifts on Soil Basic Properties

In the study area, the soil was weakly alkaline, with mean pH values of 8.8, 8.3, 8.4, and 8.5 recorded at sampling sites in SF, VFL, WL, and CF, respectively (Table 1). Notably, the pH value at SF was significantly higher than those at VFL, WL, and CF (*p* < 0.05). There were no significant differences in soil salinity among the four crop types. The average salinity values were 2.8 mg kg^−1^ at SF, 2.7 mg kg^−1^ at VFL, 0.8 mg kg^−1^ at WL, and 1.9 mg kg^−1^ at CF. Soil pH and salinity were highest at SF. Sunflowers can grow in high-pH and high-salinity environments because their deep root systems absorb soil salts during grows [22]. The electrical conductivity of the irrigation water was about 620~850 µs/cm. The lower irrigation volume at SF led to increased salt accumulation in the soil [29,30].

Regarding SOM, the average concentrations were 8.6, 13.2, 12.1, and 11.6 g kg^−1^ at SF, VFL, WL, and CF, respectively. The SOM content at SF was significantly higher than that in other crop types. The SOM levels in the study area were classified as grade IV and V, indicating a deficient level according to the nutrient grading standard of the Second National Soil Survey [31]. For TN, the mean value at SF was 0.70 g kg^−1^, significantly lower than those at VFL, WL, and CF, which were 1.03, 0.98 and 1.02 g kg^−1^, respectively. In the study area, TN levels were categorized into grade III and V, corresponding to moderate and deficient levels [31]. The lower SOM and TN at SF were due to the fact that sunflower requires less nutrients for growth [22,32]. The amounts of organic and nitrogen fertilizer applied at SF were 4557 and 268 kg hm^−2^, respectively, lower than those in other crop types (6852~8418 and 264~417 kg hm^−2^).

The soil was sandy loam. Sand was the predominant particle, accounting for 62.0%~68.7%, followed by silt (26.7%~33.5%) and clay (4.1%~5.8%). Statistical analysis showed no significant differences in particle compositions among different crop soils (*p* > 0.05). The high proportion of sand particles in the sampling area indicates good soil aeration. It also helps the soil lose salt and prevents secondary salinization during irrigation [33,34].

### 3.2. Effect of Crop Type Shifts on Soil P Storage, P Availability, and P Fractions

The TP content in the SF, VFL, WL, and CF was 848.0 ± 229.1, 975.7 ± 196.6, 878.5 ± 89.3, and 870.8 ± 128.3 mg kg^−1^, respectively (Table 2). It was classified as Grade II, indicating an abundant level (800~1000 mg kg^−1^) according to the nutrient grading standard of the Second National Soil Survey [31]. Meanwhile, the Olsen-P content in SF was relatively low. The mean Olsen-P concentration in SF was 10.3 mg kg^−1^, significantly lower than that in VFL (28.0 mg kg^−1^) and CF (14.1 mg kg^−1^), and similar to that in WL (9.8 mg kg^−1^) (Table 2). Furthermore, the CaCl_2_-P in SF, VFL, WL, and CF was 2.5 ± 3.5, 5.2 ± 4.7, 1.3 ± 0.4, and 2.2 ± 1.9 mg kg^−1^, respectively (Table 2).

**Figure 2:**
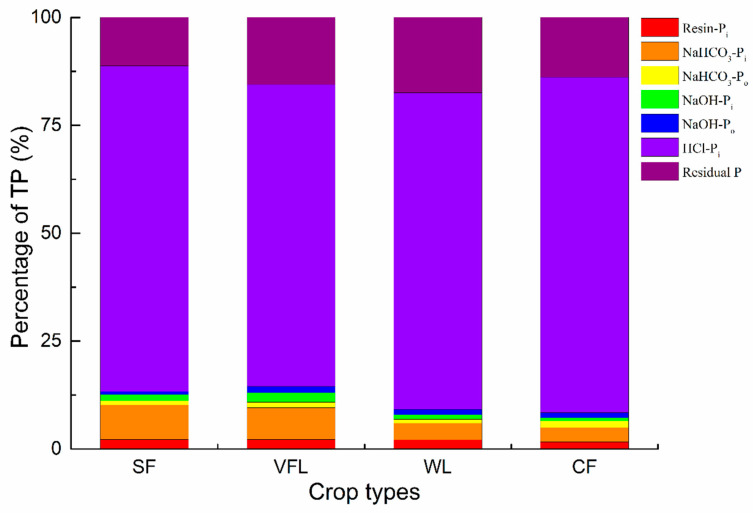
Soil phosphorus fractions at different crop types.

The seven P fractions extracted by the Hedley sequential extraction procedure can be divided into three categories: active P, moderately active P, and stable P [10,12]. Active P, considered the most biologically available P fraction, consists of Resin-Pi, NaHCO_3_-Pi, and NaHCO_3_-Po [10,12]. These components, directly accessible to plants and microorganisms, are crucial for immediate nutrient supply in the soil ecosystem [10,35]. The percentages of active P in SF, VFL, WL, and CF were 11.2%, 10.8%, 6.9%, and 6.5%, respectively. NaHCO_3_-Pi was the dominant form of active P, accounting for 3.3%~8.0%, followed by Resin-Pi (1.3%~2.2%) and NaHCO_3_-Po (0.9%~1.6%). The percentages of NaHCO_3_-Pi in SF, VFL, WL, and CF were 8.0%, 7.4%, 3.8%, and 3.8%, respectively (Figure 2). Moderately active P is typically associated with iron and aluminum oxides and easily binds to calcium, including NaOH-Pi, NaOH-Po, and HCl-Pi [10,12]. These P forms have potential availability for plant uptake and can be mobilized under specific soil conditions [12,35]. The proportions of moderately active P in SF, VFL, WL, and CF were 77.6%, 73.6%, 75.6%, and 79.6%, respectively. HCl-Pi was the predominant form of moderately active P and also of TP, accounting for 69.9%~77.7%, followed by NaOH-Pi (0.7%~2.2%), NaOH-Po (0.7%~1.4%). Residual P (Stable P) is the second-highest P component and difficult to decompose [10,12]. It represents a stable, long-term P reservoir in the soil matrix [10,35]. Although not readily available for immediate plant uptake, it has significant potential for long-term soil fertility and the slow release of P. The percentages of residual P in SF, VFL, WL, and CF were 11.3%, 15.6%, 17.5%, and 13.9%, respectively (Figure 2).

Generally, crop type shifts can impact soil P availability and P fraction [10,35]. Fertilization directly replenishes the active P pool, which decreases over time [35,36]. This decrease is due to the conversion of active P into moderately active and stable forms through processes like precipitation, adsorption, and biological retention [10,35]. After shifting to SF, TP and Olsen-P were relatively low, which was related to the lower nutrient requirements of sunflowers. The phosphate fertilizer applied in SF was 168 kg/hm^2^, lower than that in other crop types (244~267 kg hm^−2^). In SF, TP and Olsen-P were low, while the CaCl_2_-P was at a medium level, indicating a higher risk of P loss during the concentrated irrigation.

Soil acidity and alkalinity significantly influence P fixation mechanisms [15,17]. In acidic soils, P is mainly retained through ligand exchange with soil clay minerals and oxyhydroxides [15,17]. While in alkaline soils, P is more likely to be adsorbed through the precipitation of calcium phosphates and sorption on calcium carbonates and clay minerals [15,17]. In this study, VFL and WL had lower pH values than SF and CF. This lower pH increased the solubility of HCl-Pi, which was tightly bound to Ca. Since HCl-Pi is the primary P fraction in the tested region, most P was associated with Ca rather than being bound to Fe and Al oxides. Compared with other crops, SF had fewer irrigation events during its growth period. This lower irrigation frequency led to a lower utilization rate of active P and increased the risk of P loss, especially during the spring irrigation and autumn irrigation. SOM can also affect P adsorption [15,17]. Higher SOM levels can form cation bridges, increasing P adsorption sites and capacity, resulting in a higher P inventory in VFL, WL, and CF. Moreover, the difference in organic P (NaOH-Po and NaHCO_3_-Po) between different crop type soils was relatively small, suggesting that crop type conversion had a limited effect on soil organic P.

### 3.3. Soil P Leaching Change Point Calculation and P Leaching Risk Assessment

**Figure 3:**
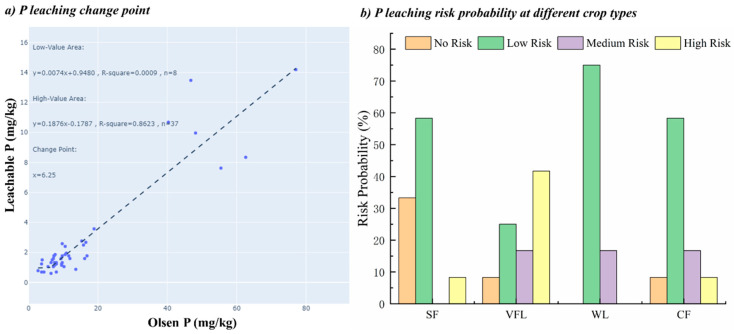
Phosphorus leaching change point and leaching risk probability measured by the SPOLERC.

The relationship between soil leachable P and Olsen-P is crucial for understanding soil P leaching risk [24,28]. According to the simulation by SPOLERC, a significant correlation was calculated between soil leachable P and Olsen-P, with a P leaching change point of 6.25 mg kg^−1^ in the sampling area (Figure 3). Heckrath et al. [37] initially identified a P leaching change point of 60 mg kg^−1^ using a linear split-line model between soil Olsen-P and dissolved reactive P in drainage water at the Broadbalk site. Subsequently, Hesketh and Brookes [38] confirmed a similar linear relationship between soil Olsen-P and 0.01M CaCl_2_ extractable P, predicting a P leaching change point within a range from 10 to 119 mg kg^−1^ across the UK. Moreover, Zhao et al. [39] reported a broader range from 30 to 160 mg kg^−1^ in Chinese agricultural soils. The soil P leaching change point has a parabolic relationship with soil pH. When pH > 6, the change point decreases as the pH increases [39]. Additionally, SOM has a high adsorption capacity for P, and an increase in SOM can raise the P leaching change point [6,39]. The lower P leaching change points in the study may be attributed to high pH and low SOM content.

The Single Factor Index (SFI) is a quantitative tool for classifying soil P leaching risk levels [10,28]. It is calculated by dividing the soil P leaching change point by soil Olsen-P content. When soil Olsen-P is below the change point (SFI ≤ 1), the P leaching risk is low [10,28]. Conversely, if soil Olsen-P exceeds the change point (SFI > 1), significant P leaching loss is expected [10,28]. The higher the SFI value is, the higher the risk level is [10,28]. Based on the P leaching change point and the SFI evaluation method, soil P leaching risk in the Hetao irrigation area can be divided into four levels: no risk (≤6.25 mg kg^−1^), low risk (6.25~12.50 mg kg^−1^), medium risk (12.50~18.80 mg kg^−1^), and high risk (≥18.80 mg kg^−1^). This classification system standardizes soil P leaching risk assessment in the Hetao irrigation area.

The probability of P leaching risk was evaluated for different land-use types based on the soil P leaching change point and Olsen-P content at sampling points [10,28]. Results showed that the P leaching risk probability varied among land-use types. The WL had the highest probability at 91.7%, followed by the VFL and CF at 83.8%, and the SF at 66.7%. A detailed risk level analysis revealed that 58.3% and 25.0% of soils in VFL and WL were medium risk and high risk, respectively, significantly higher the 8.3% and 16.7% in SF and CF. The shift in crop types have led to changes in agricultural management, such as fertilizer application and irrigation methods [10,15]. These changes affect soil P stock, availability, and soil physicochemical properties, which, in turn, contribute to P leaching risk probability [10,15].

### 3.4. Effect of Crop Type Shifts on the Abundance of Soil PSMs

The Chao1 index in SF and CF was slightly higher than that in VFL and WL. For the Shannon Index and Simpson index, the value differences were relatively small (Table 3). However, these difference in soil α-diversity among different crop types of lands were not statistically significant (*p* > 0.05; Table 3).

Analysis of the microbial community structure identified a total of 10 species of PSMs with an average relative abundance ≥0.10%, all belonging to the bacteria. Specifically, there were four species from the Actinomycetota (*Arthrobacter*, *Micromonospora*, *Streptomyces*, and *Microbacterium*), five species from the Pseudomonadota (*Bradyrhizobium*, *Escherichia*, *Mesorhizobium*, *Pseudomonas*, and *Thiobacillus*), and one from the Bacillota (*Peribacillus*). The relative abundance of Actinomycetota ranged from 4.23% to 7.81%, that of Pseudomonadota from 5.13% to 8.97%, and that of Bacillota from 0.12% to 0.31%.

**Figure 4:**
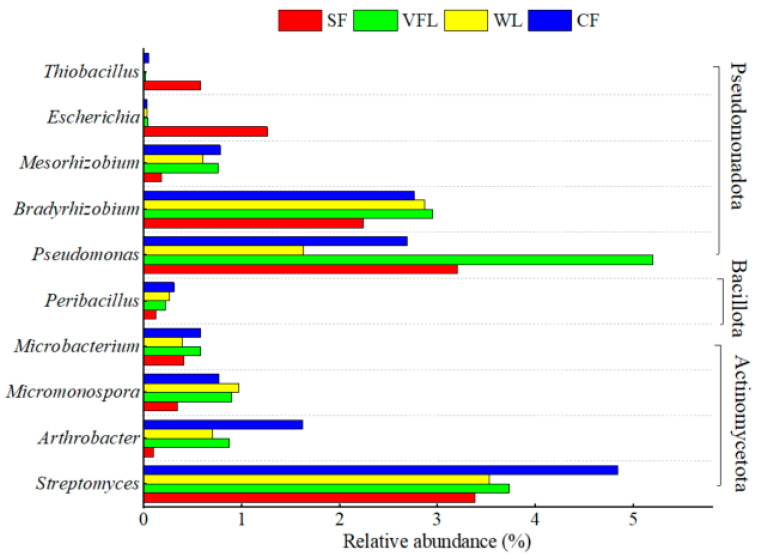
Relative abundance of soil phosphate-solubilizing microorganisms at different crop types of land.

The three dominant PSMs were *Streptomyces*, *Pseudomonas*, and *Bradyrhizobium*. The sum of relative abundances in SF, VFL, WL, and CF was 8.81%, 11.88%, 8.03%, and 10.29%, respectively. Among them, *Streptomyces* had the highest relative abundance in WL, SF, and CF, with values of 3.53%, 3.38%, and 4.87%, respectively (Figure 4). In VFL, *Pseudomonas* had the highest relative abundances (5.20%) (Figure 4). Moreover, the relative abundances of *Arthrobacter*, *Micromonospora* and *Mesorhizobium* were all above 0.5% in WL, VFL, and CF, while those of *Escherichia* and *Thiobacillus* were above 0.5% in SF (Figure 4). Bacteria, fungi, and actinomycetes can all decompose soil P [13,15,17]. Soil dephosphorylating bacteria account for 1%~50% of the total PSMs, mainly including *Bacillus*, *Pseudomonas*, *Erwinia*, and *Serratia*, etc. [13,15]. Dephosphorylating fungi only account for 0.1%~0.5% [13,15]. There are many PSMs in the rhizosphere of crops in WL and CF, while the number of saline-alkali soil is relatively low [13,15]. The types and abundances of PSMs are influenced by environmental conditions, soil type, soil physical and chemical properties, and human activities [13,15].

**Figure 5:**
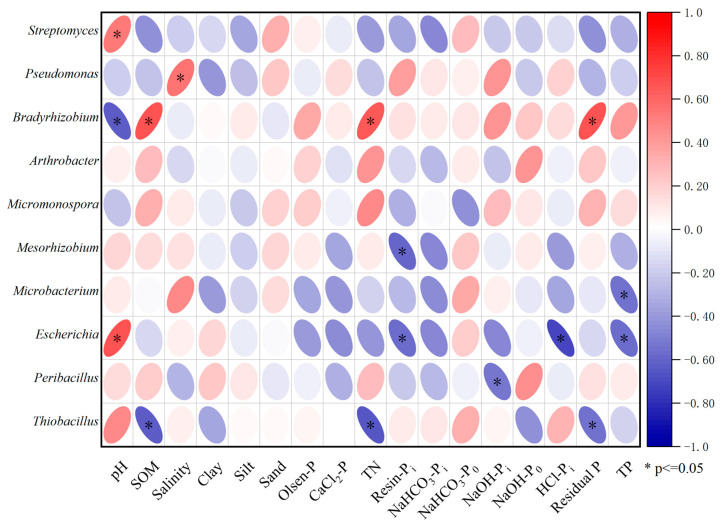
Correlation analysis between phosphorus-solubilizing microbial abundance and environmental factors.

Generally, there is a significant correlation between PSMs and soil physical–chemical properties as well as P fractions (Figure 5). Specifically, *Streptomyces* showed a significant positive correlation with soil pH (*p* ≤ 0.05). *Pseudomonas* exhibited a significant positive correlation with soil salinity (*p* ≤ 0.05). *Bradyrhizobium* had a significant negative correlation with pH and significant positive correlations with SOM, TN, and residual P (*p* ≤ 0.05). *Mesorhizobium* demonstrated a significant negative correlation with Resin-Pi (*p* ≤ 0.05). *Microbacterium* presented a significant negative correlation with TP (*p* ≤ 0.05). *Escherichia* showed a significant positive correlation with pH and significant negative correlations with Resin-Pi, HCl-Pi, and TP (*p* ≤ 0.05). *Peribacillus* had a significant negative correlation with NaOH-Pi (*p* ≤ 0.05). *Thiobacillus* exhibited significant negative correlations with SOM, TN, and residual P (*p* ≤ 0.05).

Microorganisms participate in the soil P cycle through P absorption and release, which is crucial for soil P balance and plant P uptake [13,14]. Microorganisms can immobilize P to form biomass P when there is sufficient carbon substrate. When microorganisms die, the immobilized P is released into the bio-available P pool for reuse by new microorganisms and plants [13,14]. The activation of insoluble organic and inorganic P by soil microorganisms is mainly due to bacteria and fungi [13,14]. First, increased crop P demand can shift soil microbial communities to species with higher P-solubilizing capacity [13,15]. Second, organic acids produced by soil PSMs during growth or metabolism can lower soil pH and form chelates with iron, aluminum, and other ions, blocking soil P adsorption sites and releasing plant available phosphates into the soil [13,15]. In neutral and acidic soils, organic acids dissolve and transform insoluble P through H⁺ ionization, ligand exchange, and complexation [13,15]. However, in saline-alkaline soils, the H⁺ from organic acids mainly reacts with anions like CO_3_^2−^ and HCO_3_^−^, having less impact on phosphates or phosphate ores [13,15]. Meanwhile, microorganisms can promote organic P mineralization by synthesizing phosphatases [13,15]. As highly active soil enzymes, phosphatases catalyze the hydrolysis and dephosphorylation of phospholipid bonds in SOM [40,41]. These enzymes are mainly classified as alkaline phosphatase and acid phosphatase based on their pH value [40,41]. Phosphatases can mineralize over 60% of soil organic P, thus significantly enhancing soil available P content [40,41]. Moreover, SOM is an important carbon source for soil PSMs, facilitating their growth and reproduction. Soil moisture content and aeration affect PSMs effectiveness by influencing microbial activity [2,14].

## 4. Conclusions

This research centered on the impact of crop type shift on soil phosphorus morphology and microbial functional diversity. Soils collected from a typical Hetao irrigation area, including SF, CF, WL, and VFL. Results indicated that soil pH increased significantly when other lands were converted to SF. Meanwhile, SOM, TN, and TP decreased significantly during the crop type conversion. Analysis of P fractions showed that moderately active P, including NaOH-Pi, NaOH-Po, and HCl-Pi, were the dominant fractions in the tested soils. Among them, HCl-Pi was the major component of moderately active P. The soil P leaching change point in the tested are was 6.25 mg Olsen-P kg^−1^. The probabilities of P leaching in WL, VFL, CF, and SF were 91.7%, 83.8%, 83.8%, and 66.7%, respectively. Additionally, the sum of the relative abundances of three PSMs in SF, VFL, WL and CF were 8.81%, 11.88%, 8.03% and 10.29%.

In conclusion, crop type shifts to SF intensify the soil degradation process. Soil total P and residual P content decreased. However, the content of NaHCO_3_ increased slightly, which might have been due to the rise in the abundance of *Thiobacillus* and *Escherichia*. As the cultivation area of SF expands, future research should prioritize the impacts of land-use changes, soil degradation, and reduced P reservoirs on crop yields.

## Figures and Tables

**Table 1:** The soil physical and chemical properties at different crop types.

Crop Types	pH	SOM	TN	Salinity	Sand	Silt	Clay
	-	g kg^−1^		mg kg^−1^	%		
SF *	8.8 ± 0.5 a ^†^	8.6 ± 1.8 b	0.70 ± 0.17 b	2.8 ± 4.4 a	62.2 ± 12.5 a	32.0 ± 9.4 a	5.8 ± 3.7 a
VFL	8.3 ± 0.3 b	13.2 ± 5.9 a	1.03 ± 0.40 a	2.7 ± 3.2 a	67.7 ± 11.1 a	26.7 ± 9.3 a	5.6 ± 2.5 a
WL	8.4 ± 0.1 b	12.1 ± 1.9 a	0.98 ± 0.18 a	0.8 ± 0.5 a	68.7 ± 8.9 a	27.2 ± 7.6 a	4.1 ± 2.4 a
CF	8.5 ± 0.1 b	11.6 ± 2.7 a	1.02 ± 0.18 a	1.9 ± 2.9 a	62.0 ± 10.6 a	33.5 ± 9.6 a	4.5 ± 1.8 a

* SF, sunflower field; VFL, vegetable and fruit land; WL, wheat land; CF, corn field. ^†^ Means ± SD followed by a different lowercase letter indicate significant difference from different crop types of land.

**Table 2:** Soil Olsen-P, CaCl_2_-P, and TP at different crop types.

Crop Types	Olsen-P	CaCl_2_-P	TP
	(mg kg^−1^)		
SF *	10.3 ± 11.8 ac ^†^	2.5 ± 3.5 ac	848.0 ± 229.1 a
VFL	28.0 ± 25.0 b	5.2 ± 4.7 b	975.7 ± 196.6 a
WL	9.8 ± 3.0 c	1.3 ± 0.4 c	878.5 ± 89.3 a
CF	14.1 ± 14.2 c	2.2 ± 1.9 c	870.8 ± 128.3 a

* SF, sunflower field; VFL, vegetable and fruit land; WL, wheat land; CF, corn field. ^†^ Means ± SD followed by a different lowercase letter indicate significant difference from different crop types of land.

**Table 3:** Soil α-diversity at different crop types of land.

Crop Types	Chao1 Index	Shannon Index	Simpson Index
SF *	1686 ± 327 a^†^	5.5 ± 0.8 a	0.88 ± 0.09 a
VFL	1558 ± 107 a	5.5 ± 0.1 a	0.85 ± 0.01 a
WL	1514 ± 120 a	5.1 ± 0.3 a	0.82 ± 0.01 a
CF	1770 ± 202 a	5.6 ± 0.2 a	0.85 ± 0.02 a

* SF, sunflower field; VFL, vegetable and fruit land; WL, wheat land; CF, corn field. ^†^ Means ± SD followed by a different lowercase letter indicate significant difference from different crop types of land.

## Data Availability

The datasets used and/or analyzed during the current study are available from the corresponding author on reasonable request.
